# The Royal College of Surgeons of England

**Published:** 1897-10-23

**Authors:** 


### THE ROYAL COLLEGE OF SURGEONS OF ENGLAND.

The newly-published annual calendar shows that there are 1,191 fellows, 17,333 members, 590 licentiates in midwifery, 1,186 licentiates in dental surgery, and 282 diplomates in public health on the roll. The income of the college from all sources amounted to £28,975. During the latter half of 1896, the number of specimens from the throats of diphtheria patients admitted to the hospitals of the Metropolitan Asylums Board, which were examined at the Research Laboratories, amounted to 8,701, or an average of 55·4 per diem. A large amount of anti-toxic serum of a high degree of potency had been prepared by Dr. Sims Woodhead, F.R.S., and had been distributed for use in the treatment of the disease.

